# The Application of the Omaha System in Community Rehabilitation Nursing for Patients With Stroke and Previous Falls

**DOI:** 10.3389/fneur.2022.711209

**Published:** 2022-03-31

**Authors:** Xiaoqun Fang, Shulei Jia, Qiuyan Wang, Huifang Liu, Yumei Zhou, Lingling Zhang, Tanghua Dai, Hui Luo, Hui Peng, Jun Yuan, Huyan Zhou

**Affiliations:** ^1^Department of Rehabilitation Medicine, The Second Affiliated Hospital of Nanchang University, Nanchang, China; ^2^School of Nursing, Nanchang University, Nanchang, China

**Keywords:** Omaha System, stroke, community, rehabilitation nursing, falls

## Abstract

**Objective:**

This study aimed to explore the use of the Omaha System in rehabilitation and nursing methods and the effects on patients within the community who had experienced stroke and previous falls.

**Methods:**

This study enrolled 42 patients who had experienced stroke and previous falls and had returned to the community after being discharged from the Department of Neurology and Rehabilitation of the Affiliated Hospital of Nanchang University from January to July 2018. The patients were randomly divided into two groups: an experimental group (*n* = 21) and a control group (*n* = 21). Patients in the control group received routine community rehabilitation care, and patients in the experimental group received community rehabilitation care on the basis of the Omaha System. Intervention lasted for 1 year. The Omaha outcome score, the ability to perform activities of daily living (ADL) (measured via the Modified Barthel Index [MBI]), and the incidence of falls for each group were compared before and after the intervention.

**Results:**

After 1 year of intervention, the Omaha outcome score and MBI of both groups were higher than before; the Omaha outcome score and MBI of the experimental group were higher than those of the control group; the differences were statistically significant (*P* < 0.05). No fall occurred in either of the two groups.

**Conclusion:**

The Omaha System can comprehensively evaluate the health problems of patients, guide nursing intervention, and quantitatively evaluate the effect of nursing intervention; it is therefore worthy of promotion.

## Introduction

Stroke is a term used for a group of acute cerebrovascular diseases that cause brain tissue damage, either because of the sudden rupture of blood vessels in the brain or the blockage of blood vessels so that blood cannot flow into the brain. The mortality rate is high, as is the disability rate, meaning that even when it is not fatal, this disease seriously affects quality of life (1). Although the community rehabilitation treatment of stroke in China has achieved certain positive results (2), there are still many problems, there is no systematic operative process for rehabilitation nursing (3), and no systematic nursing model has been formed (4). Nursing intervention is still mostly confined to training the patient's physical function, ignoring the environmental and psychological aspects of intervention and lacking coherence. The research team of our hospital conducted research within various communities in Nanchang city. Based on the Omaha System, community rehabilitation nursing norms applicable to stroke patients in China were established and their rehabilitation effect was evaluated. This study suggests that the Omaha System can comprehensively evaluate the health status of patients, support targeted nursing intervention, and quantitatively evaluate its effect.

## Subjects

A total of 42 stroke patients (22 males and 20 females) discharged from the Department of Neurology and Rehabilitation of the Affiliated Hospital of Nanchang University between January and July 2018 were selected as the research subjects. The age of the subjects ranged between 50 and 70 years (60.0 ± 8.9 years). The course of disease ranged from 1 month to 1 year, and patients were hospitalized for an average of about 3 months. The patients and their families received relevant health education and training in the hospital, and the patients began to enter the study as soon as they were discharged from the hospital. The patients were randomly divided into two groups: an experimental group and a control group (for each group, *n* = 21). According to the World Federation of Neurological Rehabilitation (WFNR) uses the following definitions for rehab: acute: 1–7 days; early subacute: 7 days to 3 months; late subacute: 3–6 months; chronic: > 6 months after the acute event (5). Nineteen patients were subacute, including 10 in the experimental group and 9 in the control group. Sixteen patients were subacute, including 8 in the experimental group and 8 in the control group. Seven patients were chronic, including 3 in the experimental group and 4 in the control group. The inclusion criteria were: (1) The patient had fallen after stroke in the past 6 months; (2) the patient's vital signs were stable and the disease was under control; (3) the patient's Modified Barthel Index (MBI) was below 40; (4) the patient was willing to go home for rehabilitation, and his/her family members were in agreement; and (5) the patient's family members had acquired relevant knowledge and skills after training. Patient with (1) unconsciousness; (2) incoordination or incoordinate family members; (3) receive no necessary support from their family members; (4) cognitive dysfunction (include aphasia) were excluded from the study. The differences in gender, age, level of education, occupation, and disease severity between the two groups were not statistically significant (*P* > 0.05); the two groups were therefore comparable. The Descriptive characteristics of the participants are shown in Table 1.

**Table 1:** Descriptive characteristics of the participants.

**Age** **(years)**	**Gender**	**Participants** **number**	**MBI at** **discharge**	**Course of** **Disease** **(range)** **(month)**	**Falling** **experience** **after** **stroke** **in the** **past 6** **months**
50–60	M	8	27.25 ± 1.43	[4–10]	Y
	F	7	28.56 ± 2.01	[3–12]	Y
61–70	M	10	26.25 ± 1.98	[1–13]	Y
	F	10	26.44 ± 1.87	[1–12]	Y
71–77	M	4	22.21 ± 2.11	[2–6]	Y
	F	3	21.73 ± 1.39	[3–4]	Y
Total		42			

*M, male; F, female. Y, yes; N, no*.

## Study Methods

### Research Tools

#### Research Team

The research team included three specialist rehabilitation nurses, two therapists, three community nurses, and two researchers. The specialist rehabilitation nurses and therapists each had more than 10 years of clinical work experience and were responsible for guidance and implementation. The community nurses each had more than 20 years of rehabilitation nursing experience; they received self-care-deficit rehabilitation training and were responsible for the implementation of community rehabilitation nursing. The researchers included a Master of Nursing and a nursing undergraduate who were responsible for observing and recording the implementation process of community rehabilitation nursing and for statistical analysis. All team members could skillfully use the Omaha System. One of them, a professional specialized in conducting MBI assessments, was blinded to the group of patients (experimental or control), and she was not involved in community rehabilitation care, but only in questionnaire distribution and collection.

#### The Omaha System

The Omaha System is one of the standardized nursing languages recognized by the American Nursing Association (ANA) (6). It consists of three interrelated subsystems: a problem classification scheme, an intervention scheme, and an outcome evaluation scale. The process of using it usually includes six links (7): data evaluation, statement of problem(s), confirmation of health problem score(s), nursing plan and implementation, evaluation in nursing process, and evaluation of outcomes. The Omaha nursing model first appeared in the United States, but its advanced nursing concept has now been accepted by medical staff in a variety of countries and is recognized by increasing numbers of medical practitioners (8). In China, it was mainly introduced by Professor Jinyue Huang of Hong Kong Polytechnic University, whose doctoral student, Shaoling Wang, first applied the Omaha System to the continuous nursing of patients with chronic obstructive pulmonary disease (COPD) (9, 10). It has now been applied in many fields, including community nursing, rehabilitation nursing, and nursing research (11). In 2010, the Chinese Mainland Community Nursing Training Manual compiled by the Community Health Service Cooperation Center of the World Health Organization (WHO) used the Omaha System as one of the training components for community nurses (12).

#### Effect Evaluation

**Omaha Problem Rating Scale for Outcomes** (13, 14): The problems existing in the four domains of the environment, social psychology, physiology, and health-related behaviors of patients—and the effect after intervention—were scored in the areas of cognition, behavior, and status (K-B-S) by the Likert 5-level scoring method. The scoring system is as follows:

(1) Cognition (the ability of the client to remember and understand information): 1 = lack of cognition, 2 = little cognition, 3 = basic cognition, 4 = sufficient cognition, and 5 = full cognition.
(2) Behavior (to what extent the client exhibits observable reactions, actions, or behaviors to fit a specific situation or purpose): 1 = inappropriate, 2 = rarely appropriate, 3 = occasionally appropriate, 4 = usually appropriate, and 5 = consistently appropriate.
(3) Status (how the client presents in a situation, relative to subjective and objective defining characteristics): 1 = extremely severe symptoms and signs, 2 = severe symptoms and signs, 3 = moderate symptoms and signs, 4 = mild symptoms and signs, and 5 = no symptoms and signs.

**Activities of Daily Living (ADL)** (15): The Modified Barthel Index (MBI) was used to evaluate the ability of patients to perform ADL at discharge and after 1 year of intervention. The MBI is an authoritative scale for evaluating ADL. It includes 10 activities (eating, personal hygiene, bathing, etc.), with 10 points available for each, giving a total potential score of 100 points. A score of 0–40 indicates severe dependence, a score of 41–60 indicates moderate dependence, a score of 61–99 indicates mild dependence, and a score of 100 points indicates no dependence. The higher the score, the lower the dependence and the better the self-care ability.

### Methods of Intervention

To ensure the effective implementation of community rehabilitation care, base on the guidelines for stroke prevention and treatment in China, face-to-face visits and online communication were adopted for both the experimental group and the control group. Upon discharge from the hospital, both groups of patients signed an informed consent form. The research team conducted home visits to the two groups of patients at the same times and frequency; in addition, QQ groups (a group chat tool in the instant message software QQ provided by Tencent) were established for the two groups of patients and the research team to facilitate online communication and guidance and to ensure real-time understanding of patients' health issues. After being discharged from the hospital, patients in both groups made follow-up visits to the Neurology and Rehabilitation Department according to their individual conditions. Specific guidance measures include: Cognitive interventions: face-to-face counseling, health flyers, making rehabilitation pamphlets for patients and explaining them, preaching knowledge, correcting misconceptions. Stress guidance: establishing online social platforms to communicate at all times and between patients, teaching patients and families relaxation techniques, guiding patients and families to get online support.

#### Intervention Methods for the Experimental Group

**Application of the Omaha System:** The research team designed a nursing evaluation form based on the Omaha Problem Classification Scheme and scoring scale and conducted a comprehensive assessment of patients through physical examinations to determine each patient's existing health problems in four domains: environmental, psychosocial, physiological, and health-related behaviors. Each patient provided signed informed consent at discharge. The research team provided 14 home visits for each patient (twice a month for the first 2 months after discharge, and once a month thereafter) and agreed with the patient to establish a common care goal for achieving expectations and solving health problems according to the patient's expectations and the health problems identified during each assessment. Under the guidance of this goal, the research team provided individualized education, guidance and consultation, treatment and procedures, and case management and monitoring based on the Omaha intervention program. The specific times and content of the home visits of the experimental group are shown in Table 2.

**Table 2:** Specific time and content of family visit.

**Family visit time**	**Order of home visit**	**Family visit content**
One month after discharge	1st home visit	(1)Assess the environment, and comprehensively evaluate the health problems of the patients through nursing examinations and inquiries; (2) Inquiry records of the number of falls in the past, evaluation is carried out using the ADL and quality of life questionnaire and the Omaha Outcome Evaluation System; (3) Develop nursing goals based on patient expectations and health problems; (4) Implement targeted intervention measures based on the guidance of the Omaha intervention system
Two month after discharge	2nd home visit	(1) Assessment of environmental, physical, psychological, and social issues; (2) Revise the nursing goals based on patient expectations and existing problems; (3) Implement targeted intervention measures based on the guidance of the Omaha intervention system
Three month after discharge	3rd and 4st home visit	(1) Environmental assessment and health problem assessment; (2) Revise the nursing goals with the patient; (3) Implement targeted intervention measures based on the guidance of the Omaha intervention system
One month after discharge	5st home visit	(1) Assessment of existing problems; (2) Revise nursing goals; (3) Implement targeted intervention measures based on the guidance of the Omaha intervention system
One year after discharge	14st home visit	(1) Inquiry records of the number of falls during the intervention period, evaluation is carried out using the ADL and quality of life questionnaire and the Omaha Outcome Evaluation System, compare the improvement of health problems before and after the intervention

#### Intervention Methods for the Control Group

Each patient in the control group provided signed informed consent at discharge. The research team provided home visits at the same times and frequency as for the experimental group, and the patients received routine rehabilitation care in the community for 1 year. The specific content of the visits was routine rehabilitation care, as well as the application of ADL and quality-of-life questionnaires for effect evaluation during visits 1 and 14. The visits were structured as follows: (1) Evaluate the patient; (2) make or revise nursing goals based on patient expectations and the health problems found in each assessment; and (3) implement nursing goals.

### Statistical Analysis

Data were statistically analyzed using SPSS 20.0 statistical software. Measurement data were expressed as mean ± standard deviation (*x* ± SD) and compared using analysis of variance and anova. Count data were expressed as percentages (%) and compared using a Chi-square test. *P* < 0.05 was considered statistically significant.

## Results

### Rehabilitation Nursing Framework for Self-Care Deficit of Community Patients Based on the Omaha System

Following the Omaha Problem Classification Scheme, the research team specifically analyzed the problems faced by the patients in four domains—environmental, psychosocial, physiological, and health-related behaviors—and proposed targeted intervention measures (Table 3).

**Table 3:** Rehabilitation nursing framework for community patients with self-care deficits based on the Omaha System.

**Field**	**Problem**	**Specific nursing issues**	**Intervention system**	**Score(1–5)**
Environmental field	Income Personal hygiene Housing problem	No/low income, financial difficulties Poor personal hygiene, absence of self-caring ability Insufficient security measures	Find resources and services; Train patients to comb their hair, wash their face and take a bath, etc. Renovate the living environment, purchase safety equipment, and manage water and electricity to avoid risks	K B S
Psychosocial field	Interpersonal relationship Role change Mental health	Few sharing activities Involuntary role change Melancholy/hopeless/declined self-esteem	Encourage patients to communicate with others Educate patients to correctly understand the role transformation and correctly understand the disease; Let patients bear certain responsibilities and obligations and build confidence	K B S
Physiological field	Neuro-muscle-skeletal function	Restricted range of motion, weakened muscle strength, weakened coordination, difficulty in transferring	Active/passive exercises, isometric exercises, stretching exercises and weightlifting exercises; use safety equipment correctly	K B
	Urinary system	Dysuria, urinary incontinence	Bladder function training; self-intermittent catheterization; control urinary volition, voluntary urination	
	Skin	Pressure sore, inflammation, slow healing of incision	Massage, turning over; pay attention to observe and change the dressing in time	S
	Digestion-hydration Language	Indigestion, reflux, anorexia Defects in comprehension, abnormal pronunciation	Eat a balanced diet; avoid choking; exercise properly Train patients to pronounce and use Non-verbal communication	
Health-related behaviors	Personal care	Difficulties in going to the toilet, personal hygiene, putting on and taking off clothes	Defecation function training; correct the patient's hygiene habits, from the full compensation system to partly or completely self-care; assist or guide the patient to dress;	K B
	Sleep rest pattern	Sleep pattern disorder	Ensure that patients have periodic quietness and mental states; medication	
	Nutrition	Malnutrition, insufficient intake	Evaluate nutritional status, provide a reasonable diet	S
	Others	Insufficient health care sources, Non-compliance with recommended drug dosage	Look for resources and take care by special personnel; stop drug abuse, rationally use drugs	

### Omaha Outcome Score of the Experimental Group at Discharge and After 1 Year of Intervention

In the experimental group and the control group, the K-B-S scores after 1 year of intervention according to the Omaha System were significantly improved compared with those at discharge (*P* < 0.05) (Tables 4, 5).

**Table 4:** Omaha Outcome score at discharge and at 1 year after the intervention in the experimental group.

**Time**	**Environment**			**Psychosocial field**			**Physiological field**			**Health-related behaviors**		
	**Cognition**	**Behavior**	**Status**	**Cognition**	**Behavior**	**Status**	**Cognition**	**Behavior**	**Status**	**Cognition**	**Behavior**	**Status**
At discharge	1.69 ± 0.52	1.15 ± 0.13	1.27 ± 0.73	1.76 ± 0.84	1.75 ± 0.63	2.73 ± 0.33	1.75 ± 0.65	1.73 ± 0.29	1.73 ± 0.22	1.73 ± 0.18	1.73 ± 0.13	1.14 ± 0.78
One year of intervention	4.27 ± 0.22	4.55 ± 0.27	4.10 ± 0.34	3.36 ± 0.48	3.79 ± 0.34	3.73 ± 0.57	3.63 ± 0.83	3.76 ± 0.38	3.78 ± 0.82	3.82 ± 0.68	4.13 ± 0.62	4.19 ± 0.36
*F*	54.31	58.45	53.76	41.42	49.23	21.49	47.47	49.69	48.42	50.54	53.07	55.42
*P*	<0.01	<0.01	<0.01	<0.01	<0.01	<0.01	<0.01	<0.01	<0.01	<0.01	<0.01	<0.01

**Table 5:** Omaha Outcome score at discharge and at 1 year after the intervention in the control group.

**Time**	**Environment**			**Psychosocial field**			**Physiological field**			**Health-related behaviors**		
	**Cognition**	**Behavior**	**Status**	**Cognition**	**Behavior**	**Status**	**Cognition**	**Behavior**	**Status**	**Cognition**	**Behavior**	**Status**
At discharge	1.54 ± 0.62	1.20 ± 0.20	1.17 ± 0.49	1.65 ± 0.76	1.67 ± 0.54	2.61 ± 0.60	1.69 ± 0.58	1.64 ± 0.31	1.69 ± 0.34	1.65 ± 0.20	1.65 ± 0.09	1.21 ± 0.62
One year of intervention	2.53 ± 0.54^b^	2.22 ± 0.49^b^	1.98 ± 0.90^b^	2.19 ± 0.50^b^	2.41 ± 0.40^b^	2.99 ± 0.49^b^	2.35 ± 0.74^b^	2.83 ± 0.21^b^	2.41 ± 0.70^b^	2.44 ± 0.72^b^	2.51 ± 0.54^b^	2.52 ± 0.46^b^
*F*	33.12	32.54	39.43	41.32	37.37	12.93	32.73	31.99	37.54	39.45	38.45	37.68
*P*	<0.01	<0.01	<0.01	<0.01	<0.01	<0.01	<0.01	<0.01	<0.01	<0.01	<0.01	<0.01

*Compared with the experimental group after 1 year of intervention.*

b *P < 0.05*.

### Comparison of Activities of Daily Living Score and Quality of Life Between the Two Groups at Discharge and After 1 Year of Intervention

There were significant differences between the MBI (used to evaluate ADL) before and after intervention in each of the two groups (*P* < 0.05). Although there was no significant difference in MBI between the two groups (*P* > 0.05) at discharge, there was significant difference between the two groups after 1 year of intervention. In this regard, the MBI score of the experimental group was higher than that of the control group after 1 year of intervention according to the Omaha System (*P* < 0.05) (Table 6).

**Table 6:** Comparison of MBIs at discharge and 1 year after intervention in the two groups (points, *x* ± SD).

**Group**	**Number**	**At discharge**	**One year of intervention**
Experimental group	21	26.45 ± 2.56	54.63 ± 4.65^a^^b^
Control group	21	25.81 ± 2.53	39.61 ± 2.79^a^

*Compared within the group at discharge.*

a *P < 0.05; Compared with the control group after 1 year of intervention.*

b *P < 0.05*.

### Incidence of Falls

No fall occurred in any patient in either the experimental group or the control group during 1 year of intervention, i.e., the incidence of falls was 0% (*P* < 0.05).

## Discussion

### Effective Application of a Community Rehabilitation Nursing Model for Stroke Patients Based on Omaha System Theory

This model is based on the Omaha System and Maslow's hierarchy of needs. The specific health problems of the experimental group during 1 year after discharge are regarded as the clinical basis, the Omaha Problem Rating Scale for Outcomes is regarded as the basis for evaluation, and a rehabilitation nursing framework for self-care deficit in community patients is established. In this study, the K-B-S Omaha outcome scores of the experimental group in the environmental, psychosocial, physiological, and health-related behaviors domains were significantly higher than the scores at discharge (*P* < 0.05). This result reveals that when the Omaha System is successfully applied, it can promote the community rehabilitation of stroke patients. The highest grades after improvement was seen in the environmental domain, in which all patients scored 4 or 5 (out of 5); the health-related behaviors domain had the highest improvement range (4–5); and the psychosocial domain had the lowest improvement range (3–4). This reveals that given the guidance of professionals and their support in and implementation, stroke patients and their family members have sufficient cognition to improve and maintain environmental conditions as much as possible and usually appropriately modify and avoid risky behaviors, although minor problems or symptoms still occur. Meanwhile, the psychosocial domain is the weak link in the comprehensive rehabilitation of stroke patients (16). A study conducted in China revealed that of the stroke survivors surveyed, 93.5 and 62.7% needed psychological support and social support, respectively (17). One scholar clearly points out that there is a need to focus on psychological and social factors in the community rehabilitation process in China (18). Depression is a common mental symptom after stroke, with an incidence of 5–67% (19). One study reported that depression in stroke patients in the rehabilitation stage could affect the frequency of social activities and the possibility of social participation, finding a negative correlation (20). In this study, patients did not exhibit the same level of improvement in the psychosocial domain as in the other fields, suggesting that stroke patients need long-term and sustained social and psychological support.

### Effective Improvement of the Self-Care Ability of Stroke Patients

The results of the present study reveal that the application of either the Omaha System or conventional community rehabilitation nursing can improve the self-care ability of stroke patients within the community. In patients receiving intervention under the Omaha System (i.e., the treatment group), the score for self-care ability (MBI) after 1 year was significantly improved from discharge; the score of this group was higher than that of patients receiving conventional rehabilitation nursing (i.e., the control group) (*P* < 0.05). There is consistency between the results of the present study and those of Yanlin Wang's study (21): There, too, the application of either the Omaha System or routine nursing was able to effectively improve the self-care ability of stroke patients, and the score (MBI) of the experimental group was higher than that of the control group. Community rehabilitation nursing can promote the health of stroke patients (9). In this study, the ADL score (MBI) of the control group was improved after 1 year of intervention, but the patients were still at the stage of severe dependence (0–40 points); in comparison, the patients in the experimental group, with intervention carried out according to the Omaha System, achieved an improvement within the year from severe dependence (0–40 points) at discharge to moderate dependence (41–60 points). Therefore, the application of the Omaha System can increase the effectiveness of intervention to improve the self-care ability of stroke patients.

### Contribution to More Comprehensive Nursing Evaluation and the Implementation of Holistic Nursing

The Omaha System nursing evaluation method was more comprehensive and effective in application than the traditional evaluation method (22), and the use of the Omaha System structured the process of community rehabilitation nursing and promoted the implementation of holistic nursing. The Omaha Problem Classification Scheme includes four domains: environmental, psychosocial, physiological, and health-related behaviors. The subjects of problems can be individuals, families, or communities, can involve health promotion, and can be existing or potential, covering all aspects of the biopsychosocial medical model. Compared with the traditional nursing model, in which nursing is given based on the practitioner's own experience and knowledge and guided by the patient's expectations, community nurses applying the Omaha System understand, comprehensively evaluate, and identify patients' health problems in a timely way according to the characteristics of the Omaha Problem Classification Scheme. Having identified existing or potential nursing problems, they describe them accurately in unified and standardized nursing language; then, targeted problem-based nursing is implemented. The evaluation–intervention–evaluation framework of the Omaha System drives nurses to dynamically understand the health status of each patient and the intervention effect. In its application, the Omaha System guides nurses to focus on patients' health problems by comprehensively considering their physiological, psychosocial and health-related behaviors, as well as environmental domains and other factors, and to implement feasible nursing measures. Finally, the effect is quantitatively evaluated, and nursing is completed systematically, scientifically, and directionally.

### Improvement of the Fall Situation of Stroke Patients

Stroke is the third leading cause of persistent disability globally. Stroke patients are almost twice as likely to fall as other people of the same age and gender, and falls after stroke may lead to injury, pain, fear of falls, maladjustment, and increased care costs (23). In this study, no fall occurred in any patient in either the experimental group or the control group. A prior study revealed that the fall rate in stroke patients was high during the community rehabilitation period and that the most common cause of falls was the loss of balance when walking indoors (24). The maintenance of human balance mainly depends on three factors: sensory input, central integration, and motor control (25). In this study, the self-care ability of both the experimental group and the control group was improved after 1 year (*P* < 0.05). The improvement of self-care ability means that patients can carry out ADL, with or without the help of others, through having a sense of the outside world, integrating information via the central nervous system, and controlling their muscles. Making daily attempts to carry out ADL also forms training for patients on sensory input, central integration, and motor control.

### Limitations and Prospects

The first two limitations of this study involve size and duration. The sample size in this study was small: Each group followed up for 1 year after discharge contained only 21 subjects. Although the various studies carried out by Chinese scholars on the application of the Omaha System have differed in duration, there has not yet been a long-term follow-up study. The reason may be cost related—studies of longer duration incur greater costs. Further research is needed to study the permanent effect of the Omaha System, including long-term research into community rehabilitation nursing.

Additionally, this study ignored the cost–benefit correlation and did not carry out a cost analysis. Due to a shortage of funds in the community, the professional team in this study provided free family visits and nursing. It is hoped that in order to achieve the better and wider development and application of community rehabilitation nursing, the community will pay attention to and support community rehabilitation nursing in the future and that family visits will be included in the social insurance system to alleviate the economic difficulties involved in the implementation of this service.

Finally, although professionals were involved in the community rehabilitation nursing in this study, the cooperation of patients and their families was also necessary, and there were variations in compliance and cooperation between the different patients and/or their families. Although the Omaha score is quite high, the patient's daily activities still need others' help from the indications of the MBI (used to evaluate ADL). The reason for this contradiction may be due to the different standards among assessors, and more stringent standardized training and quality control are needed in future research. Moreover, in this study, only the Omaha outcome score and MBI of the both group were examined and the process effect and influencing factors were not quantitatively analyzed. While patients rely on family visits and the remote supervision and guidance of professionals during the rehabilitation nursing period, it is also necessary to mobilize family members. It is important that family members cooperate in supervising and witnessing patients' progress; they need to be enabled to recognize and believe patients' problems, cooperate with patients in their improvement, and supervise them to keep them healthy.

## Data Availability Statement

The original contributions presented in the study are included in the article/supplementary material, further inquiries can be directed to the corresponding author.

## Ethics Statement

The studies involving human participants were reviewed and approved by the Second Affiliated Hospital of Nanchang University Ethics Committee. The patients/participants provided their written informed consent to participate in this study. Written informed consent was obtained from the individual(s) for the publication of any potentially identifiable images or data included in this article.

## Author Contributions

XF and SJ conceived the idea and conceptualized the study. QW and HLi collected the data. YZ, LZ, and TD analyzed the data. HLu and HP drafted the manuscript. JY and HZ reviewed the manuscript. All authors read and approved the final draft.

## Funding

This work was supported by Natural Science Foundation of Jiangxi Province (20192BBG70015), Science and technology project of Jiangxi Health Commission (202130353), Natural Science Foundation of Jiangxi Province (20171BAB215027), and Educational Reform Project of Jiangxi Province (JXJG-18-1-31).

## Conflict of Interest

The authors declare that the research was conducted in the absence of any commercial or financial relationships that could be construed as a potential conflict of interest.

## Publisher's Note

All claims expressed in this article are solely those of the authors and do not necessarily represent those of their affiliated organizations, or those of the publisher, the editors and the reviewers. Any product that may be evaluated in this article, or claim that may be made by its manufacturer, is not guaranteed or endorsed by the publisher.
